# Creating a Theoretically Grounded, Gamified Health App: Lessons From Developing the Cigbreak Smoking Cessation Mobile Phone Game

**DOI:** 10.2196/10252

**Published:** 2018-11-29

**Authors:** Elizabeth A Edwards, Hope Caton, Jim Lumsden, Carol Rivas, Liz Steed, Yutthana Pirunsarn, Sandra Jumbe, Chris Newby, Aditi Shenvi, Samaresh Mazumdar, Jim Q Smith, Darrel Greenhill, Chris J Griffiths, Robert T Walton

**Affiliations:** 1 Centre for Primary Care and Public Health Blizard Institute, Bart’s and The London School of Medicine and Dentistry Queen Mary University of London London United Kingdom; 2 Asthma UK Centre for Applied Research Bart’s and The London School of Medicine and Dentistry Queen Mary University of London London United Kingdom; 3 Faculty of Science Engineering Computing Kingston University London United Kingdom; 4 MRC Integrative Epidemiology Unit University of Bristol Bristol United Kingdom; 5 School of Psychological Science University of Bristol Bristol United Kingdom; 6 Social Science Research Unit University College London London United Kingdom; 7 Centre for Complexity Science University of Warwick Coventry United Kingdom; 8 Department of Statistics University of Warwick Coventry United Kingdom

**Keywords:** smoking cessation, health behaviors, behavioral medicine, games for health, mHealth, eHealth

## Abstract

**Background:**

Gaming techniques are increasingly recognized as effective methods for changing behavior and increasing user engagement with mobile phone apps. The rapid uptake of mobile phone games provides an unprecedented opportunity to reach large numbers of people and to influence a wide range of health-related behaviors. However, digital interventions are still nascent in the field of health care, and optimum gamified methods of achieving health behavior change are still being investigated. There is currently a lack of worked methodologies that app developers and health care professionals can follow to facilitate theoretically informed design of gamified health apps.

**Objective:**

This study aimed to present a series of steps undertaken during the development of Cigbreak, a gamified smoking cessation health app.

**Methods:**

A systematic and iterative approach was adopted by (1) forming an expert multidisciplinary design team, (2) defining the problem and establishing user preferences, (3) incorporating the evidence base, (4) integrating gamification, (5) adding behavior change techniques, (6) forming a logic model, and (7) user testing. A total of 10 focus groups were conducted with 73 smokers.

**Results:**

Users found the app an engaging and motivating way to gain smoking cessation advice and a helpful distraction from smoking; 84% (62/73) of smokers said they would play again and recommend it to a friend.

**Conclusions:**

A dedicated gamified app to promote smoking cessation has the potential to modify smoking behavior and to deliver effective smoking cessation advice. Iterative, collaborative development using evidence-based behavior change techniques and gamification may help to make the game engaging and potentially effective. Gamified health apps developed in this way may have the potential to provide effective and low-cost health interventions in a wide range of clinical settings.

## Introduction

### The Health App Revolution

Mobile phone use is increasing rapidly in both developed and developing countries, and by 2020, 70% of the world’s population will be using mobile phones [1,2]. Three billion people globally currently use mobile health apps [3-5], with over 165,000 health apps available worldwide [2].

Health apps are in high demand. A recent study reported 800,000 downloads per month of smoking cessation apps worldwide [6]; there were 400 smoking cessation apps available on app stores in 2013 when Cigbreak development began [6]. However, most health apps are not developed from a theoretical basis that draws on evidence-based behavior change techniques (BCTs), and there is little evidence that public health practitioners or users have participated in design [6-10]. To date, there have been few rigorous evaluations of the effectiveness of health apps [2,11-14] although pilot studies and small trials have shown promising results [9,11,13,15,16].

### Using Gamification to Change Health Behavior

Maintaining users’ engagement with health apps is not easy, with 77% of apps going unused only 72 hours after being installed [10,17,18]. One potential solution to this problem is gamification, which aims to harness the motivational power of *gaming elements* such as badges, leaderboards, competitions, rewards, and avatars to increase user engagement and hence improve effectiveness [2,19]. Gamification shares key elements with established health BCTs and behavior change theory [20,21], and there is growing evidence that gamification increases engagement with health apps [10,18,22,23]. Despite this, a recent review found that only 4% of top-rated health apps on the Apple and Android stores made use of gamification principles [2].

### Health App Development

In recent years, standards have been established for health app development [24], and there are some examples of well-developed health apps that are evidence- and theory-based with expert design [9,13,15,25,26]. The British Standards Institution has formulated a code of practice for health and wellness apps, providing app developers with quality criteria to consider during the development process [24]. However, there is a lack of worked methodologies that app developers and health care professionals can follow to aid development and incorporation of appropriate features.

### The Cigbreak App

In 2013, a group of clinicians, researchers, and game developers set out to build a dedicated smoking cessation app *Cigbreak*, developed in collaboration with potential end users. Gamification and theoretically validated BCTs were included, including those shown to be beneficial in smoking cessation [27,28], with the goal of creating an engaging, scientifically grounded health app.

**Figure 1:**
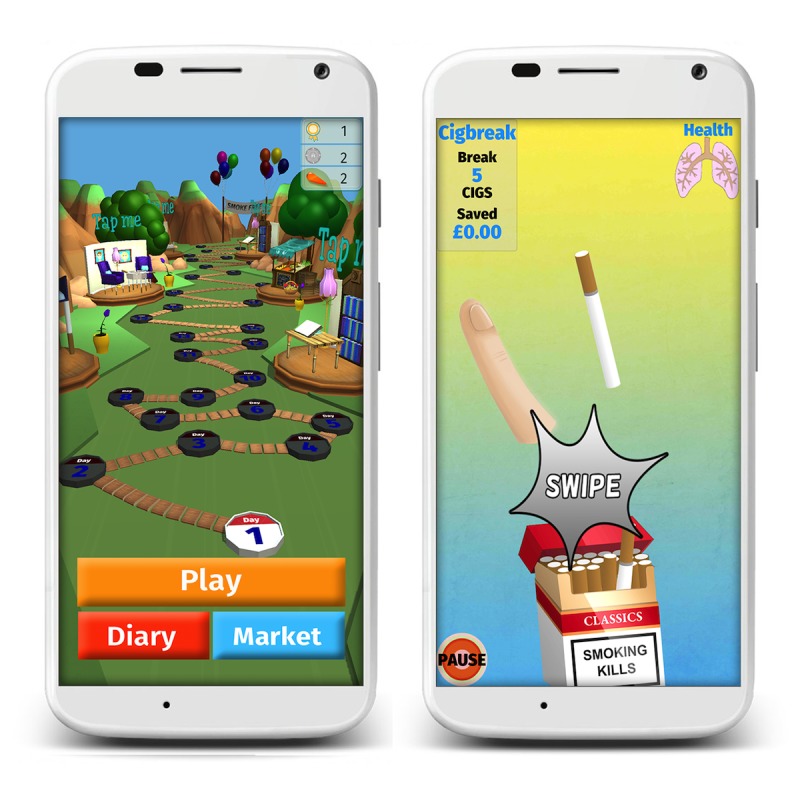
Screenshots of Cigbreak.

The current version of Cigbreak involves players swiping their screen to break cigarettes as they dance upward from a generic cigarette pack, providing a distraction from cravings. Smokers progress along a path through a garden to a smoke-free finish line (Figure 1). Along the way, players can complete diary entries and overcome specific daily missions. Players are rewarded for both real-life smoking cessation behavior and progress through the game with health messages, coins, and trophies and are given personalized feedback. Player’s progress can be monitored and shared on Facebook, providing social support. Nicotine replacement therapy (NRT) power-ups inform players of the different kinds of NRT available, encouraging pharmacological support.

This paper documents the development process in a series of steps, providing a worked example for future development of gamified health apps.

## Methods

### Agile Development Process

Principles of agile development were adopted [29] where prototypes were developed rapidly and systematically modified according to user feedback. The methods adopted in Steps 1 to 7 are described below. We aimed first to gain an understanding of the research problem (why do people still smoke?) and to identify key user preferences for app functions. Doctors, health psychologists, and researchers worked closely with app developers to incorporate established smoking cessation methods and validated BCTs. Feedback from users was obtained following each iteration to refine app functions or features. At all stages of development, the focus groups involved participants using actual prototypes of the app, having hands-on experience on mobile handsets that we provided in the 10 focus groups.

### Focus Groups

Focus groups were held throughout the development process (step 2 and step 7). Participants were recruited through pharmacies in Tower Hamlets, London; Eurogamer (Europe’s largest gaming show); a community development charity in East London, Social Action for Health; Kick-it the smoking cessation scheme facilitated through the Tri-borough Council, London. Moreover, 10 focus groups were conducted in total with 73 participants (male [n=34] and female [n=39]). In total, 2 focus groups were conducted at Chapel Hill, University of North Carolina, to understand the differences in smoking/ cessation culture between the United Kingdom and the United States, which might affect design requirements when making the app available globally. Furthermore, of the 10 focus groups, 3 (n=26) were conducted during early development as documented in step 2. There was a wide range of ages (15 to 67 years) and ethnicities (Bengali, Turkish, Russian, Polish, British, American, or African-American) represented in the focus groups, which included smokers who may find it difficult to access standard smoking cessation services (NRT and behavioral support [individual and group]), for example, routine and manual workers and adolescents.

All focus groups were conducted over a 90-min period and led by a trained facilitator (EAE) and assisted by 1 other team member (HC, LS, CR). Participants were asked to use the app for a 30-min period. The remaining 60 min were used for discussion. A basic structure was followed; however, content was also guided by participants. Questions in the first 3 focus groups differed and focused more on participants’ smoking behaviors and ideas for smoking cessation app content. The later focus groups focused more on providing user feedback on the various iterations. Participants were also asked to complete a questionnaire at the end of each focus group. Tobacco dependence was measured using the Fagerström test for nicotine dependence [30,31], and participants were asked about any current treatment they were using to help them stop smoking. Questions were also asked regarding the appearance of the app, enjoyability, and usability, and participants were asked if they would recommend the app to a friend.

Participants were given £20 to show appreciation for their time. Voice recordings of the focus groups were taken and transcribed, and observational notes were made. Thematic analysis of the data used a framework approach [32,33]. A trained researcher (EAE) independently analyzed the focus group transcripts; however, any uncertainties were discussed with the study team. We did not double code because we were coding for concrete themes to aid app development rather than conceptual themes; the second researcher in each focus group was able to check themes against their own perceptions. Moreover, 5 interconnected stages were followed in the analysis: (1) familiarization with the data, (2) identifying thematic frameworks for themes arising, (3) indexing—highlighting relevant quotes, (4) charting—arranging the quotes under themes, and (5) mapping and interpretation [32-34]. Furthermore, focus groups were conducted until a saturation period was reached in which no other new themes emerged. No software was used to conduct coding or identify themes.

#### Step 1: Forming an Expert Multidisciplinary Design Team

A multidisciplinary design team with appropriate experience and expertise was formed to aid in the design and construction of the app. The team included a senior app developer (HC), computer scientists (YP and DG), clinical doctors (EAE and RTW), a senior health psychologist (LS), a senior medical sociologist (CR), and several smoking cessation advisors from the London Kick-it smoking cessation scheme. The membership of the design team was consistent over the development period. Extensive input from the public and potential app users was sought throughout development.

The team brainstormed extensively before any coding began, to establish key concepts and constructs to be included in the app. The design team met once per week throughout the development period. Meetings were chaired by a facilitator (RTW), and decisions were documented and logged.

#### Step 2: Defining the Problem and Establishing User Preferences

First, we set out to define the problem, exploring factors influencing nicotine addiction and successful quit attempts, and we then explored user preferences with our main goal to establish app components required to create user engagement, resulting in a smoking cessation app likely to be retained by users. In total, 3 focus groups with 26 smokers were conducted.

#### Step 3. Incorporating the Evidence Base

Cigbreak development was guided by the Medical Research Council framework for complex interventions [35] following the British Standard Institute Code of Practice for Health and Wellness app development [24]. The latest guidelines for effective smoking cessation were also considered [36-39].

Cigbreak was designed with 30 game levels, which were estimated to take an average user 28 days to complete. Evidence suggests that being abstinent for 28 days increases the chance of successfully quitting [9], and hence Cigbreak can support its users through the most critical period of their smoking cessation attempt.

#### Step 4: Integrating Gamification

There are several taxonomies or frameworks of gamification available [21,40-43]; however, there is a recognized lack of high-quality studies [22] and only 1 taxonomy has been validated [40,44]. Given the lack of empirical guidance in this area, we relied on the experience of our expert multidisciplinary design team aiming to create an engaging and entertaining game.

Having gamified the app, we then turned to one of the more popular taxonomies to deconstruct Cigbreak in an effort to understand how the game might address various aspects of motivation and to optimize features. Cugleman and colleagues identified 7 key gamification strategies: goal setting, capacity to overcome challenges, providing feedback on performance, reinforcement, comparing progress, social connectivity, and fun and playfulness [21].

#### Step 5: Incorporating Behavior Change Techniques

A BCT is “an observable, replicable and irreducible component of an intervention designed to alter or redirect causal processes that regulate behavior; that is, a technique is proposed to be an ‘active ingredient’ (eg, feedback, self-monitoring, reinforcement)” [45]. BCTs have been clearly defined and classified into an internationally recognized taxonomy [45]. It was understood that if our intervention was to be effective, it would be important to make good use of BCTs. Therefore, in addition to providing distraction from cravings, the game would become part of a causal pathway for health behavior change.

This development phase of Cigbreak involved close collaboration with a health psychologist, who advised on BCTs on the basis of strong empirical evidence for their efficacy [27,28] and feedback gained in earlier focus groups. A systematic review of BCTs in existing health apps was also conducted [2]. Self-regulatory BCTs (*feedback & monitoring* including *self-monitoring of behavior*) have been commonly used in gamified apps to promote physical activity, healthy eating, and alcohol reduction [2,12,46,47]. These specific BCTs were effective in achieving behavior change in previous studies [48-53], and therefore, they were an obvious choice for inclusion.

#### Step 6: Developing a Logic Model and Investigating Causal Pathways

A logic model to describe the program theory behind the intervention was formulated, and methods of representing and examining the operation of the intervention in mathematical models were researched.

#### Step 7: User Testing

Cigbreak was built in *Unity for Android* [54] by Healthy Games Ltd. The use of a preexisting game engine facilitated rapid development of app prototypes, which could be circulated among the team and focus groups of smokers.

## Results

### Defining the Problem and Establishing User Preferences

Personal experiences, concerns about ill health, financial pressures, and family/friends were key motivational themes for stopping smoking. Environmental factors and mood played an important role in whether smokers continued to smoke. Smokers identified a need for extra support and distraction from the action of smoking in certain environments. As their thoughts at these times were overwhelmingly related to smoking, a game including cigarettes that involved *swiping* them was felt to be helpful. It was this key finding together with brainstorming in the team that led us to develop the initial concept that the app could be used as a distraction from cravings. These focus groups drove inclusion of many other features such as giving information on health and finances and using family members to provide motivational cues. The key themes identified in motivational factors are shown in Table 1.

Smokers perceived that health information including benefits of quitting and harms of smoking was important. However, smokers preferred more focus on positive outcomes and emotions rather than negative. Smokers felt that personalization was an important feature, including ability to set personal quit dates, plans, record relapses, and receive tailored text messages. The idea of a personalized diary to incorporate these aspects was popular among the smokers as were links to local pharmacies/quit services.

The appearance of the app was identified as an important factor, with smokers wanting a bright, colorful app that was interactive and entertaining. Several smokers felt that gaming elements would aid in this goal.

At the conclusion of this phase, there was a good understanding of user preferences, and the appearance of the app was beginning to take shape. The challenge was then to deliver an app that met user requirements in addition to using evidence-based smoking cessation advice and BCTs. The key themes identified for important features for a smoking cessation app are shown in Table 2.

### Integrating Gamification

Game elements were incorporated by game developers and computer scientists (Table 3) [21].

### Incorporating Behavior Change Techniques

**Figure 2:**
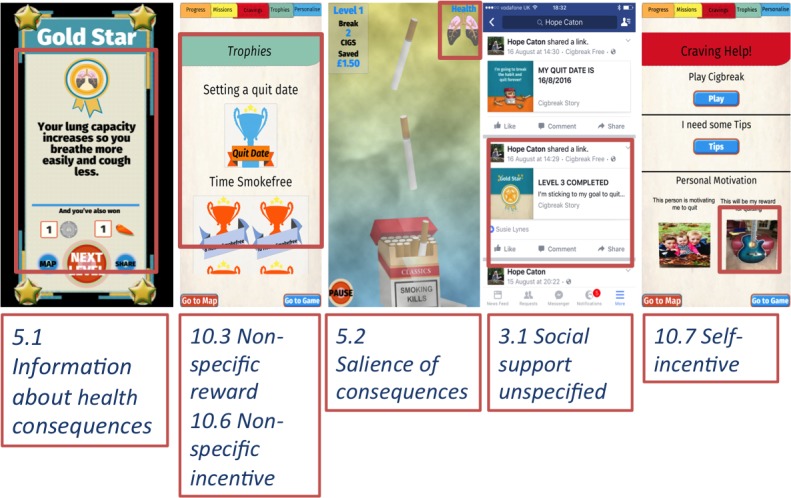
Example of embedded behavior change techniques in Cigbreak.

At the end of this phase, our design for Cigbreak included 36 BCTs (see Figure 2 and Table 4 for a full list). Two researchers trained in BCT coding (EAE and JL) coded Cigbreak independently in accordance to the (V1) BCT taxonomy [45].

Screenshots of the Cigbreak app are shown with examples of BCTs highlighted. Note some screenshots may show multiple BCTs, which have not been highlighted but would have been coded accordingly.

**Table 1:** Key themes identified in motivational factors affecting smoking and quitting.

Themes	Subthemes	Quotes
Financial	Financial factors are an important motivational factor in quitting	*If I’m short of money one month, that definitely stops me smoking.*
Mood	Stress/boredom/unhappiness contribute to motivation to smoke	*I smoke when I am stressed.*
Family and friends	Family, especially children, can be a motivation to quit	*Children can be powerful.*
Experience of illness and health concerns	Personal experiences of ill health secondary to smoking in family/friends would affect motivation to quit	*When my mum was unwell with her chest, it definitely made me think of stopping.*
Environmental factors	More likely to lapse if socializing may need more support during these activities; Job stops people smoking during the day time; Being on holiday encourages people to smoke more; Alcohol encourages people to smoke more; Government bans have been very useful	*I would smoke if others smoked around me.* *Interestingly when you know smoking not a possibility don’t really get cravings e.g. on 10 hour flight.*

**Table 2:** Key themes identified for important features for a smoking cessation app.

Themes	Subthemes	Quotes
Health information	To know the harms of smoking and the benefits of quitting	*I think what you need to hear is that stern image you have on the back of cigarettes of what it is doing to you.* *Maybe it could give you an explanation of how your health is improving.*
Distraction from craving	To keep your hands busy; To help you know what to do when you get cravings; To have a craving button	*I am craving so idea of a craving button which could be pressed when needed and links to a page with different management strategies e.g. play game, prompt to go for walk, speak to friend.*
Personalization or related to real time quitting	To be personalized to the individual; To receive personalized texts and messages at times of craving; To be able to enter personalized quit date and plan; To be able to record relapses	*Being personalized is definitely important.* *Maybe you could put dates in for your stop date.* *If you had a relapses you could put it in there too, so it is tied to your real time quitting.* *You could have it so when you wake up it says good morning this is your 11th day, that would be motivating, that would make me stop smoking.* *Maybe you could set it so it gave you little motivations reminders at times that you need them.*
Bright in color	To be bright in color	*Being colorful is important.*
Interactive	To be interactive	*It has to be interactive to work.*
Gamification	To contain gaming elements; To be fun	*Having a game and having to do something with your hands does work because you will spend an hour on that game before you have realized it and that an hour you have not smoked a cigarette.* *Games are very good, candy crush is brilliant, I was playing it at 3am when I wanted a cigarette, doing something quick with your hands takes your mind off it.*
Diary	To contain a personal dairy	*Like for example I want to do something in my diary, like today I have stopped and I really fell like a cigarette, so I would write down I have saved x amount by not going out and buying cigarettes.*
Financial saving	To contain information on the financial savings of quitting	*I would like to know I have saved x amount by not going out and buying cigarettes* *After a week if would be good to tot up how much money you have saved.*
Links to local services, pharmacies	Links to local quit smoking services or phone lines or local pharmacies	*That would really help, or it could give locations of stop smoking places.*
Information about NRT^a^	To contain information about different NRT products available and how to use them	*I would like to know more about the different products available.*
Emotional content	To have an emotional content, would prefer focus on positive not negative emotions; To promote feelings of relaxation and happiness	*It needs to have an emotional content, but I think more focus on the positive rather than the negative.* *I want it to help me relax.*

^a^NRT: nicotine replacement therapy.

**Table 3:** Gamification strategies in Cigbreak, including their location in the app.

Gamification strategy	Description	Location in the app
Goal setting	Players are asked to set or agree on goals and to set a quit date	Diary
Capacity to overcome challenges	The diary contains missions and challenges, for example calling a quit line or contacting their local pharmacy for advice on using approved medications; The game involves players increasing speed and avoiding objects when slicing cigarettes	Diary; Game
Feedback	Players are given feedback on outcomes of their behavior, for example, the number of days smoke-free and number of cigarettes smoked; Players are provided with a score for number of cigarettes sliced	Diary; Game
Reinforcement	Players are rewarded with trophies for setting quit date and for not smoking	Diary; Game
Comparing progress	Players are asked to share their scores with their friends on Facebook. Friends can compare scores and trophies	Game; Diary
Social connectivity	Players can connect with friends for social support via Facebook	Diary; Game
Fun and playfulness	Players break cigarettes that dance upward from a generic cigarette pack while trying to avoid breaking any vegetables. Players are rewarded with gold, silver, and bronze stars for breaking the required number of cigarettes	Game

**Table 4:** Behavior change techniques (BCTs) in Cigbreak with location in the game.

BCT	Description	Location in the game
1.1 Goal setting (behavior)	Players are asked to set goals, for example, a quit date	Diary
1.2 Problem solving	Players are asked to identify specific triggers that generate the urge to smoke and develop strategies for avoiding environmental triggers	Diary
1.3 Goal setting (outcome)	Players are asked to set goals, for example, to save money as a result of the money saved from not smoking to pay for a holiday	Diary
1.6 Discrepancy between current behavior and goal	If players continue to smoke after their quit date, they are provided with feedback on the discrepancy between current smoking and their previously set outcome goals	Diary
2.2 Feedback on behavior	Players are given feedback on their behavior, for example, number of cigarettes smoked since starting the app	Diary
2.3 Self-monitoring of behavior	Players are asked to monitor their smoking daily in a diary in the app. Players are also asked daily in pop-up questions the number of cigarettes smoked and whether players have remained smoke-free	Diary
2.7 Feedback on outcomes of behavior	Players are given feedback on the number of days smoke-free and what this means for their health	Diary
3.1 Social support (unspecified)	Players are asked to share their scores and smoke free status with their friends on Facebook	Game
3.2 Social support practical	Players are provided with practical advice in the diary on how to gain support from friends or colleagues or staff to help them to quit; for example, players are provided with information on support from community pharmacies	Diary
4.2 Information about antecedents	Players are prompted in the diary to record situations or circumstances in which they are more likely to get cravings or experience the urge to smoke, for example, being at a bus stop	Diary
5.1 Information about health consequences	Players are provided written, audible, and visual information on the health consequences of smoking and the health benefits of quitting; Health reward badges inform of the health consequences of smoking	Game
5.2 Salience of consequences	The avatar’s lungs become blackened and cough as cigarettes are not destroyed, thus highlight damage to lungs from not stopping	Game
5.3 Information about social and environmental consequences	Players are provided with written and visual information regarding social and environmental consequences of smoking	Game
5.6 Information about emotional consequences	Players are provided with written information regarding emotional consequences of quitting smoking, for example, that quitting smoking increases happiness and life satisfaction	Game
7.1 Prompts or cues	Players are prompted to play the game at times of cravings via the crave button	Game or Diary
8.2 Behavior substitution	Players are prompted to substitute smoking with an alternative behavior; For example, players are prompted to play the game via the crave button when they have a craving to smoke or prompted to go for a walk	Diary
8.7 Graded tasks	Tasks or missions set to help players to quit smoking become increasingly more difficult, but achievable, until players have quit	Diary
9.1 Credible source	The app is cocreated by doctors, health psychologists, and smoking cessation advisors. Players are made aware of this on the app store, website, and in the app	Diary
9.2 Pros and cons	Players are asked to list the pros and cons for quitting smoking in the diary	Diary
9.3 Comparative imagining of future outcomes	Players are asked to imagine and compare possible outcomes following quitting smoking, imagining themselves as nonsmokers and document this in the diary	Diary
10.3 Nonspecific reward	Players are provided with trophies, stars, and virtual coins for staying smoke-free	Game
10.4 Social reward	Players are congratulated by friends on social media or Facebook	Game or Diary
10.6 Nonspecific incentive	Players are incentivized to stay smoke-free	Game
10.7 Self-incentive	Players are encouraged to plan to reward themselves in the future for staying smoke free	Diary
10.9 Self reward	Players are encouraged to reward themselves at the present time if they have stayed smoke free	Diary
10.10 Reward (outcome)	Players are provided with in-game rewards for remaining smoke-free; for example, they are provided with trophies and health reward badges	Game
11.1 Pharmacological support	Players are encouraged to use NRT^a^ and to obtain this from their pharmacy. There are also NRT power-ups in the game, which allow players to progress to higher levels in the game. This encourages the use of NRT and also informs players of the different NRT available	Game
11.2 Reduce negative emotions	Players are given advice on reducing negative emotions to quit smoking; for example, using stress management skills	Diary
12.1 Restructuring the physical environment	Players are advised how to change their physical environment to help themselves to quit, for example to remove cigarettes from their home and to remove ash trays	Diary
12.2 Restructuring the social environment	Players are advised and given tips on how to change their social environment to help them to quit, for example, avoiding contact with friends who smoke	Diary
12.3 Avoidance or reducing exposure to cues for the behavior	Players are advised and given tips on how to avoid exposure to specific social and contextual or physical cues with regard to smoking, for example, avoiding pubs and bars that have been associated with smoking	Diary
12.4 Distraction	The gameplay involves players breaking cigarettes acting as a distraction from smoking	Game
12.5 Adding objects to the environment	Players are provided with the app and written or visual information to aid smoking cessation	Diary
13.5 Identity associated with changed behavior	Players are advised to construct or articulate a new self-identity as an ex-smoker	Diary
15.1 Verbal persuasion about capability	Players are encouraged that they can quit smoking, arguing against self-doubts and asserting that they can and will succeed	Diary
15.3 Focus of past success	Players are asked to list previous successes, for example, when they have resisted smoking	Diary

^a^NRT: nicotine replacement therapy.

### Developing a Logic Model and Investigating Causal Pathways

**Figure 3:**
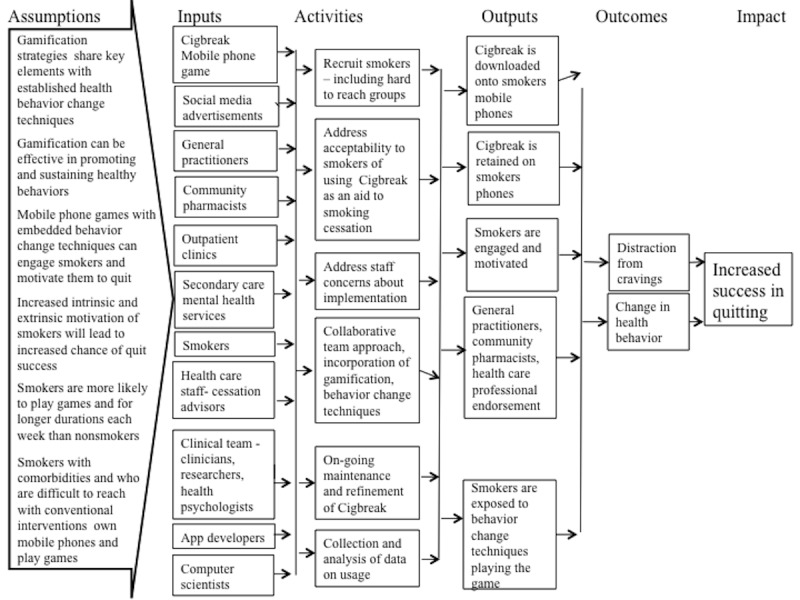
Cigbreak logic model describing the intended operation of the intervention.

The logic model (Figure 3) describes basic assumptions on which the intervention is based, together with channels through which smokers are invited to use the app, for example, social media advertisements and recommendations by clinicians. The recruitment methods are designed to reach people who find conventional health care services difficult to access.

The app is designed to record user engagement with BCTs and subsequent changes in smoking behavior to allow detailed temporal analysis of causal pathway assumptions. Frameworks and graphical models for exploring causal relations are well established [55-57], and causal methodologies to account for confounding by covariates are now sufficiently developed to enable effects of new public health interventions to be evaluated from observational studies [58-67]. The relatively unexplored approach of the Cigbreak app for improving public health holds exciting possibilities to form a defining example for the development of testing of causal assumptions and pathway analysis suited to app design. This would provide the framework for a more refined analysis of complex interventions with a dynamic longitudinal treatment regime resulting in long-term health benefit.

### User Testing

#### Changes to Cigbreak From Direct User Feedback

Key themes were identified in a framework analysis (Table 5), and the app was redesigned accordingly. For example, players wanted the app to be more personalized. In response, a personalized diary for players was introduced, which allowed players to set personal goals and quit dates and to add pictures of the item or person motivating them to quit. At this stage of app development, the thematic analysis was oriented toward more concrete app features and design elements rather than abstract concepts.

#### From Prototype to Product

Players found the prototype app to be an engaging and motivating way to deliver smoking cessation advice, providing a useful distraction from smoking (Table 6), and 84% (62/73) said they would play again or recommend to a friend. Those with higher tobacco dependence as defined by the Fagerström test for nicotine dependence [30,31] and difficult-to-reach smokers appeared to be more engaged. Engagement was measured as both flow (focused attention and enjoyment) and usage (frequency, duration, and depth of usage) [18]. Players said they would be happy to obtain this app from their pharmacy or general practitioner.

The game was completed in 2015 with funding from the London Tri-borough Smokefree Alliance and is commissioned in 5 London boroughs. Following the final focus groups, Cigbreak was released on both Apple and Google Play stores in January 2016.

**Table 5:** Key themes identified by Cigbreak users to aid app development.

Theme	Subthemes	Quotes
Health	Players wanted more emphasis on health gains; Players wanted more hard health facts and graphic imagery; Players wanted the characters’ lungs to be linked to reality	*I want to know the other benefits.* *I like the lungs in the corner but they need to be linked to reality. I need fear to motivate me.*
Personalization	Players wanted the game to be more personalized and wanted a personalized avatar	*I want the game to be personalised to me.* *I like the character in the corner, but I want to personalise him.*
Goals	Players wanted goal setting linked to real life behavior	*I want to go on holiday so my goal is to save £x.*
Levels or graphics	Players wanted more variation in the levels; Players wanted the game to become increasingly more difficult; Players wanted more interactive graphics; Players wanted brighter colors and graphics	*Needs more variation to keep you interested.* *It needs to get harder and harder and needs to be more engaging.* *Needs to be more interactive and enticing.* *Needs to be brighter in color.*
Financial	Players wanted the financial rewards more related to real life	*It would be good to see what I have saved per week.*

**Table 6:** Key themes identified using Cigbreak as a smoking cessation tool.

Themes	Subthemes	Quotes
Distraction	Players felt the game would work as a distraction	*I think it could work, candy crush saga was my life when I was quitting, so if it was related to smoking that would be even better.* *It’s a good distraction.* *It definitely stops me thinking of cigarettes.*
Cravings	Players felt the app could be used at times of cravings	*I would pick this up and play when I had a craving.* *I could use this at the bus stop when I get cravings.*
Availability	Players would be happy to obtain from their pharmacy or GP^a^	*Every GP should know about it, to give it to their patients.*

^a^GP: general practitioner.

## Discussion

### Principal Findings

A qualitative approach working directly with the target population at an early stage was key to the design process. Feedback on early prototypes helped us to gain an understanding of the problem [24], establish user preferences, and ensure desired functions were included [24,68].

Forming an expert development team was vital to ensure that our app was evidence based and potentially effective [69]. Computer scientists and app developers were included to implement game elements and health psychologists, clinicians, and researchers for incorporation of BCTs. Careful consideration of which BCTs to include was needed, with a clear understanding of which techniques are theoretically linked to the required mechanisms of action [70].

It was found that including imagery of cigarettes and smoking-related cues did not act as a trigger to smoke. Feedback from the qualitative work suggested that a smoking-related game would be more engaging compared with a nonsmoking–related game as attentional bias is toward smoking when craving occurs. The literature also supports this finding. A randomized controlled trial compared Nicot, a video game in which players crush virtual cigarettes in a 3D game environment, with a balloon popping game. Nicot was found to improve smoking cessation rates by 13% [70]. Sanders-Jackson and colleagues found along with Due and colleagues, Hogarth and colleagues, and Meinke and colleagues that individuals who have chemical addictions are more likely to attend visually to objects associated with their addiction [71-74].

It was found that participants wanted to have positive rather than negative messaging and imagery. Gain-framed messages shift smoking-related beliefs, attitudes, and behaviors toward the direction of avoidance and cessation [75,76]. However, Mayes and colleagues found that a combination of both might be beneficial, specifically in younger smokers [77]. Moorman and Putte found that a positive frame is preferable when nicotine dependence or quitting intention is lower, and conversely, a negative frame worked better when nicotine dependence and quitting intention are both high [78].

### Comparison With Prior Work

There are currently no published frameworks for production of gamified health apps with systematic inclusion of BCTs or use of behavior change theory.

Several nongamified health apps use an iterative user-centered design process [9,13,79-136]. Most apps are designed by an expert multidisciplinary team. However, only 3 explicitly used agile development [137-140], although this form of technology development is gaining in popularity, and it is anticipated that there will be greater use in the future.

There are several published frameworks for developing nongamified health apps [26,139,141-147]. The Schnall and colleagues framework uses the Information Systems Research framework [141,148]. The Goyl and colleagues framework [143] combines knowledge to action [149] and the MRC framework for complex interventions [33]. The Tombor and colleagues framework [26] is also guided by MRC framework for complex interventions and Multiphase Optimization Strategy [35,150]. Moreover, 6 frameworks also consider the incorporation of behavior change theory. Curtis and colleagues adopted the behavior change wheel (BCW) [144,151]. Tombor and colleagues also uses the BCW and the BCT taxonomy version 1 [26,45,151]. Wilhide and colleagues consider behavioral models [142], whereas Goyal and colleagues and Whittaker and colleagues incorporate social cognitive theory [143,146,152]. Mummah and colleagues combine use of both theory and a taxonomy [145]. Of the frameworks, 3 do not incorporate any form of behavior change theory [139,141,147].

As this area of research is still nascent, it is unclear if any of the above approaches are superior to the approach adopted. The framework recommend is based on our experience in having a successfully developed gamified health app rather than empirical evidence. Recent frameworks including our own are designed around guidelines for complex interventions involving a systematic development process with an iterative user-centered approach based on theory and evidence. It is likely that these elements are key for the development of health apps that may be effective at modifying health behaviors.

### Strengths and Limitations

Although there are smoking cessation apps that incorporate gamification techniques, to date, there is no publicly available dedicated game to promote smoking cessation or guidance for the development of gamified health apps. Raiff and colleagues have developed a prototype smoking cessation mobile phone game using the concept of contingency management (delivering rewards contingent on objective evidence of smoking abstinence) with virtual in-game rewards and social connectivity. The prototype game differs from Cigbreak in concept and mechanic, with the prototype game involving swiping pollen-gems, not including imagery of cigarettes or smoking- related cues. In addition, 71% of participants in a small usability study reported that the program would help them personally to quit smoking [153]. The prototype has not yet been released on app stores and is still in development.

In this study, an example is provided of a systematic approach for developing a gamified smoking cessation app with systematic use of BCTs and evidence-based practice guided by user input. This could provide a potential methodology for development of other gamified health apps. However, the framework proposed has not been evaluated, and thus it cannot be concluded that apps developed using this framework are superior or that the methods adopted are superior to other methods. Future work could include a comparison of usability or acceptability outcomes and behavior change outcomes for apps developed using this framework compared with apps that have not used this framework. The rapidly changing technological landscape and change in sophistication of users may limit the applicability of the findings to future app development.

There are further limitations to the methods adopted. Focus groups can be subject to group bias; however, in turn, small focus groups can generate a natural open discussion, providing fruitful feedback as to usability. Participants were given different iterations of the app to use during the focus groups, which may potentially have biased participants to focus on the functionality of the app presented to them. However, this is a natural consequence of agile iterative development. Only 1 trained researcher independently analyzed the focus group transcripts, which may have led to bias; however; any uncertainties found were discussed with the study team.

Although Cigbreak has been made available to the public on a small scale, the app has not yet been formally evaluated against clinical endpoints. A rigorous evaluation to assess impact on short- and long-term quit rates is being planned.

### Implications for Clinicians and Policy Makers

Mobile phone games could provide a potentially cost-effective platform for health promotion and thus have a substantial public health impact. However, developers of digital interventions need to adhere to existing regulatory frameworks and emerging standards [24,69] to develop games that health practitioners can feel confident to recommend to patients.

Questions remain as to the best way to evaluate health apps in this rapidly changing field, without stifling innovation. Michie and colleagues present recent consensus from experts at an international workshop on digital interventions in relation to health behaviors, concluding that evaluations should be made during all phases of the development cycle and need not rely solely on traditional methods [69]. New experimental methods and adaptive research designs such as A/B testing and N-of-1 studies may be used to make best use of rich data streams and assess outcomes within shorter time frames [69]. There is considerable scope for using emerging methods of analyzing observational data in this context [60].

### Unanswered Questions and Future Research

Further work is needed to identify potential causal pathways and mechanisms in health apps in general and specifically for Cigbreak. Existing methods such as structured equation modeling and pathway analysis can be applied to study short- to medium-term effects such as Cigbreak engagement. However, current methods only explore assumed causal relations between limited numbers of variables and are not well adapted to complex models or digital interventions. Thus, there are exciting possibilities for future development of more refined analysis of applications and for generating ecologically valid, real-time objective data.

Game analytics provide a rich source of data, and machine learning techniques can be used to make changes to the game design to improve the users’ experience and potentially to modify health outcomes. The key is to ensure that the correct metrics are captured during gameplay and then to use the techniques to identify patterns in the data. It should then be possible to change gameplay adaptively to optimize some chosen criteria. These methods could be used to change health behavior if this is being captured as one of the metrics. Bauckhage and colleagues [154] discuss a number of the methods in clustering game behavior data, and use of such techniques in Cigbreak is currently being explored. Future research also aims to ascertain which components of a multicomponent intervention such as Cigbreak are accounting for what effects.

## Abbreviations

BCT: behavior change technique
BCW: behavior change wheel
NRT: nicotine replacement therapy

## References

[1] Lunden I (2015). Techcrunch.

[2] Edwards EA, Lumsden J, Rivas C, Steed L, Edwards LA, Thiyagarajan A, Sohanpal R, Caton H, Griffiths CJ, Munafò MR, Taylor S, Walton RT (2016). Gamification for health promotion: systematic review of behaviour change techniques in smartphone apps. Br Med J Open.

[3] García-Gómez JM, de la Torre-Díez I, Vicente J, Robles M, López-Coronado M, Rodrigues JJ (2014). Analysis of mobile health applications for a broad spectrum of consumers: a user experience approach. Health Informatics J.

[4] Cusano D (2015). Telecareaware.

[5] (2018). Statistia.

[6] Abroms LC, Lee WJ, Bontemps-Jones J, Ramani R, Mellerson J (2013). A content analysis of popular smartphone apps for smoking cessation. Am J Prev Med.

[7] Boulos MN, Wheeler S, Tavares C, Jones R (2011). How smartphones are changing the face of mobile and participatory healthcare: an overview, with example from eCAALYX. Biomed Eng Online.

[8] Hoeppner B, Hoeppner Susanne S, Seaboyer Lourah, Schick Melissa R, Wu Gwyneth W Y, Bergman Brandon G, Kelly John F (2016). How Smart are Smartphone Apps for Smoking Cessation? A Content Analysis. Nicotine Tob Res.

[9] Ubhi HK, Michie S, Kotz D, Wong WC, West R (2015). A mobile app to aid smoking cessation: preliminary evaluation of SmokeFree28. J Med Internet Res.

[10] Perski O, Blandford A, Ubhi HK, West R, Michie S (2017). Smokers' and drinkers' choice of smartphone applications and expectations of engagement: a think aloud and interview study. BMC Med Inform Decis Mak.

[11] Zhao J, Freeman B, Li M (2016). Can mobile phone apps influence people's health behavior change? An evidence review. J Med Internet Res.

[12] Crane D, Garnett C, Brown J, West R, Michie S (2015). Behavior change techniques in popular alcohol reduction apps: content analysis. J Med Internet Res.

[13] Garnett C, Crane D, Michie S, West R, Brown J (2016). Evaluating the effectiveness of a smartphone app to reduce excessive alcohol consumption: protocol for a factorial randomised control trial. BMC Public Health.

[14] Haskins BL, Lesperance D, Gibbons P, Boudreaux ED (2017). A systematic review of smartphone applications for smoking cessation. Transl Behav Med.

[15] Bricker JB, Mull KE, Kientz JA, Vilardaga R, Mercer LD, Akioka KJ, Heffner JL (2014). Randomized, controlled pilot trial of a smartphone app for smoking cessation using acceptance and commitment therapy. Drug Alcohol Depend.

[16] Buller DB, Borland R, Bettinghaus EP, Shane JH, Zimmerman DE (2014). Randomized trial of a smartphone mobile application compared to text messaging to support smoking cessation. Telemed J E Health.

[17] Chen A (2017). New data shows losing 80% of mobile users is normal, and why the best apps do better.

[18] Perski O, Blandford A, West R, Michie S (2017). Conceptualising engagement with digital behaviour change interventions: a systematic review using principles from critical interpretive synthesis. Transl Behav Med.

[19] King D, Greaves F, Exeter C, Darzi A (2013). 'Gamification': influencing health behaviours with games. J R Soc Med.

[20] Cheek C, Fleming T, Lucassen MF, Bridgman H, Stasiak K, Shepherd M, Orpin P (2015). Integrating health behavior theory and design elements in serious games. JMIR Ment Health.

[21] Cugelman B (2013). Gamification: what it is and why it matters to digital health behavior change developers. JMIR Serious Games.

[22] Johnson D, Deterding Sebastian, Kuhn Kerri-Ann, Staneva Aleksandra, Stoyanov Stoyan, Hides Leanne (2016). Gamification for health and wellbeing: A systematic review of the literature. Internet Interv.

[23] Hamari J (2014). Does gamification work?--a literature review of empirical studies on gamification.

[24] British Standards Institution (2015). Health and wellness apps.Quality criteria across the life cycle. Code of Practice.

[25] Rose K, Koenig M, Wiesbauer F (2013). Evaluating success for behavioural change in diabetes via mHealth and gamification: MySugr's keys to retention and patient engagement. Diabetes Technol.

[26] Tombor I, Shahab L, Brown J, Crane D, Michie S, West R (2016). Development of SmokeFree Baby: a smoking cessation smartphone app for pregnant smokers. Transl Behav Med.

[27] West R, Walia A, Hyder N, Shahab L, Michie S (2010). Behavior change techniques used by the English Stop Smoking Services and their associations with short-term quit outcomes. Nicotine Tob Res.

[28] Michie S, Churchill S, West R (2011). Identifying evidence-based competences required to deliver behavioural support for smoking cessation. Ann Behav Med.

[29] Flood D, Chary A, Austad K, Diaz AK, García P, Martinez B, Canú WL, Rohloff P (2016). Insights into global health practice from the agile software development movement. Glob Health Action.

[30] Fagerström K (2012). Determinants of tobacco use and renaming the FTND to the Fagerstrom Test for Cigarette Dependence. Nicotine Tob Res.

[31] Fagerstrom K, Schneider NG (1989). Measuring nicotine dependence: a review of the Fagerstrom Tolerance Questionnaire. J Behav Med.

[32] Pope C, Ziebland S, Mays N (2000). Qualitative research in health care. Analysing qualitative data. Br Med J.

[33] Rabiee F (2004). Focus-group interview and data analysis. Proc Nutr Soc.

[34] Ritchie J (1994). Qualitative data analysis for applied policy research. In Analysing Qualitative Data.

[35] Craig P, Dieppe P, Macintyre S, Michie S, Nazareth I, Petticrew M, Medical Research Council Guidance (2008). Developing and evaluating complex interventions: the new Medical Research Council guidance. Br Med J.

[36] US Public Health Service (2008). A clinical practice guideline for treating tobacco use and dependence: 2008 update. Am J Prev Med.

[37] (2014). National Centre for Smoking Cessation and Training. Local Stop Smoking Services: Service and delivery guidance 2014.

[38] (2015). Smoking: reducing and preventing tobacco use.

[39] West R, McNeill A, Raw M (2000). Smoking cessation guidelines for health professionals: an update. Health Education Authority. Thorax.

[40] Quick JM, Atkinson RK, Lin L (2012). Empirical taxonomies of gameplay enjoyment: personality and video game preference. Int J of Game Based Learn.

[41] Hunicke R, LeBlanc M, Zubek R (2004). MDA: A Formal Approach to Game Design and Game Research.

[42] King D, Delfabbro P, Griffiths M (2009). Video game structural characteristics: a new psychological taxonomy. Int J Ment Health Addiction.

[43] Ferdig RE (2018). Handbook of Research on Effective Electronic Gaming in Education (Three Volume Set).

[44] Quick J, Atkinson R, Lin L (2012). Confirming the taxonomy of video game enjoyment. http://www.academia.edu/19611557/Proceedings_of_the_Games_Learning_Society_Conference_Vol._4.

[45] Michie S, Richardson M, Johnston M, Abraham C, Francis J, Hardeman W, Eccles MP, Cane J, Wood CE (2013). The behavior change technique taxonomy (v1) of 93 hierarchically clustered techniques: building an international consensus for the reporting of behavior change interventions. Ann Behav Med.

[46] Direito A, Dale LP, Shields E, Dobson R, Whittaker R, Maddison R (2014). Do physical activity and dietary smartphone applications incorporate evidence-based behaviour change techniques?. BMC Public Health.

[47] Conroy DE, Yang C, Maher JP (2014). Behavior change techniques in top-ranked mobile apps for physical activity. Am J Prev Med.

[48] Michie S, Ashford S, Sniehotta FF, Dombrowski SU, Bishop A, French DP (2011). A refined taxonomy of behaviour change techniques to help people change their physical activity and healthy eating behaviours: the CALO-RE taxonomy. Psychol Health.

[49] Michie S, Churchill S, West R (2011). Identifying evidence-based competences required to deliver behavioural support for smoking cessation. Ann Behav Med.

[50] Michie S, Abraham C, Whittington C, McAteer J, Gupta S (2009). Effective techniques in healthy eating and physical activity interventions: a meta-regression. Health Psychol.

[51] Greaves CJ, Sheppard KE, Abraham C, Hardeman W, Roden M, Evans PH, Schwarz P, IMAGE Study Group (2011). Systematic review of reviews of intervention components associated with increased effectiveness in dietary and physical activity interventions. BMC Public Health.

[52] Dombrowski S, Sniehotta Ff, Avenell A, Johnston M, MacLennan G, Araújo-Soares V (2012). Identifying active ingredients in complex behavioural interventions for obese adults with obesity-related co-morbidities or additional risk factors for co-morbidities: a systematic review. Health Psychology Review.

[53] O'Brien N, McDonald Suzanne, Araújo-Soares Vera, Lara Jose, Errington Linda, Godfrey Alan, Meyer Thomas D, Rochester Lynn, Mathers John C, White Martin, Sniehotta Falko F (2015). The features of interventions associated with long-term effectiveness of physical activity interventions in adults aged 55-70 years: a systematic review and meta-analysis. Health Psychol Rev.

[54] Unity Unity User Manual (2018.1).

[55] Pearl J (2009). Causality.

[56] Pearl J (2009). Causal inference in statistics: an overview. Statist Surv.

[57] Spirtes P, Glymour C, Scheines R (2001). Causation, Prediction, and Search.

[58] Rubin DB (2004). Direct and indirect causal effects via potential outcomes. Scand J Stat.

[59] Rubin DB (2005). Causal inference using potential outcomes. J Am Stat Assoc.

[60] Dawid A (2002). Influence diagrams for causal modelling and inference. Int Stat Rev.

[61] Pearl J (1995). Causal diagrams for empirical research. Biometrika.

[62] Shpitser I, Pearl J (2008). Complete identification methods for the causal hierarchy. Journal of Machine Learning Research.

[63] Thwaites P, Smith JQ, Riccomagno E (2010). Causal analysis with chain event graphs. Artif Intell.

[64] Kline R (2015). Principles and Practice of Structural Equation Modeling, Fourth Edition (Methodology in the Social Sciences).

[65] Pearl J, Hoyle R (2012). The Causal Foundations of Structural Equation Modeling. Handbook of Structural Equation Modeling.

[66] Robins JM (1999). Association, causation, and marginal structural models. Synthese.

[67] Robins JM, Hernán MA, Brumback B (2000). Marginal structural models and causal inference in epidemiology. Epidemiol.

[68] Michie S, Yardley Lucy, West Robert, Patrick Kevin, Greaves Felix (2017). Developing and Evaluating Digital Interventions to Promote Behavior Change in Health and Health Care: Recommendations Resulting From an International Workshop. J Med Internet Res.

[69] (2006). WK Kellogg Foundation.

[70] Raiff BR, Jarvis BP, Rapoza D (2012). Prevalence of video game use, cigarette smoking, and acceptability of a video game-based smoking cessation intervention among online adults. Nicotine Tob Res.

[71] Sanders-Jackson AN, Cappella JN, Linebarger DL, Piotrowski JT, O'Keeffe M, Strasser AA (2011). Visual attention to antismoking PSAs: smoking cues versus other attention-grabbing features. Hum Commun Res.

[72] Due L, Huettel SA, Hall WG, Rubin DC (2002). Activation in mesolimbic and visuospatial neural circuits elicited by smoking cues: evidence from functional magnetic resonance imaging. Am J Psychiatry.

[73] Hogarth L, Dickinson A, Hutton SB, Elbers N, Duka T (2006). Drug expectancy is necessary for stimulus control of human attention, instrumental drug-seeking behaviour and subjective pleasure. Psychopharmacology (Berl).

[74] Meinke A, Thiel CM, Fink GR (2006). Effects of nicotine on visuo-spatial selective attention as indexed by event-related potentials. Neuroscience.

[75] Cataldo J, Hunter M, Petersen AB, Sheon N (2015). Positive and instructive anti-smoking messages speak to older smokers: a focus group study. Tob Induc Dis.

[76] Schneider T, Salovey P, Pallonen U, Mundorf N, Smith NF, Steward WT (2001). Visual and auditory message framing effects on tobacco smoking. J Appl Social Pyschol.

[77] Mays D, Turner MM, Zhao X, Evans WD, Luta G, Tercyak KP (2015). Framing pictorial cigarette warning labels to motivate young smokers to quit. Nicotine Tob Res.

[78] Moorman M, van den Putte B (2008). The influence of message framing, intention to quit smoking, and nicotine dependence on the persuasiveness of smoking cessation messages. Addict Behav.

[79] Sobrinho A, da Silva LD, Perkusich A, Pinheiro ME, Cunha P (2018). Design and evaluation of a mobile application to assist the self-monitoring of the chronic kidney disease in developing countries. BMC Med Inform Decis Mak.

[80] Baron KG, Duffecy J, Reid K, Begale M, Caccamo L (2018). Technology-assisted behavioral intervention to extend sleep duration: development and design of the Sleep Bunny mobile app. JMIR Ment Health.

[81] Wang J, Yao NA, Liu Y, Geng Z, Wang Y, Shen N, Zhang X, Shen M, Yuan C (2017). Development of a Smartphone Application to Monitor Pediatric Patient-Reported Outcomes. Stud Health Technol Inform.

[82] Cheng C, Lee L, Cheng Y (2017). Design and evaluation on the mobile application of Transcutaneous Electrical Nerve Stimulation (TENS). Stud Health Technol Inform.

[83] Tonkin E, Jeffs L, Wycherley TP, Maher C, Smith R, Hart J, Cubillo B, Brimblecombe J (2017). A smartphone app to reduce sugar-sweetened beverage consumption among young adults in Australian remote indigenous communities: design, formative evaluation and user-testing. JMIR Mhealth Uhealth.

[84] Davis S, Peters D, Calvo RA, Sawyer SM, Foster JM, Smith L (2017). “Kiss myAsthma”: using a participatory design approach to develop a self-management app with young people with asthma. J Asthma.

[85] Castensøe-Seidenfaden P, Reventlov Husted G, Teilmann G, Hommel E, Olsen BS, Kensing F (2017). Designing a self-management app for young people with type 1 diabetes: methodological challenges, experiences, and recommendations. JMIR Mhealth Uhealth.

[86] Danilovich MK, Diaz L, Saberbein G, Healey WE, Huber G, Corcos DM (2017). Design and development of a mobile exercise application for home care aides and older adult medicaid home and community-based clients. Home Health Care Serv Q.

[87] Halsall V, Rogers J, Witt J, Song S, Nguyen HD, Kelly P (2017). Development of a mobile app for family planning providers. MCN Am J Matern Child Nurs.

[88] Cai R, Beste D, Chaplin H, Varakliotis S, Suffield L, Josephs F, Sen D, Wedderburn LR, Ioannou Y, Hailes S, Eleftheriou D (2017). Developing and evaluating JIApp: acceptability and usability of a smartphone app system to improve self-management in young people with juvenile idiopathic arthritis. JMIR Mhealth Uhealth.

[89] Werner-Seidler A, O'Dea B, Shand F, Johnston L, Frayne A, Fogarty AS, Christensen H (2017). A smartphone app for adolescents with sleep disturbance: development of the Sleep Ninja. JMIR Ment Health.

[90] Henry J, Thielman E, Zaugg T, Kaelin C, Choma C, Chang B, Hahn S, Fuller B (2017). Development and field testing of a smartphone “App” for tinnitus management. Int J Audiol.

[91] Petersen M, Hempler NF (2017). Development and testing of a mobile application to support diabetes self-management for people with newly diagnosed type 2 diabetes: a design thinking case study. BMC Med Inform Decis Mak.

[92] Pauwels K, Aerts S, Muijzers E, De Jaegere E, van Heeringen K, Portzky G (2017). BackUp: development and evaluation of a smart-phone application for coping with suicidal crises. PLoS One.

[93] Rodrigues A, Sniehotta FF, Birch-Machin MA, Olivier P, Araújo-Soares V (2017). Systematic and iterative development of a smartphone app to promote sun-protection among holidaymakers: design of a prototype and results of usability and acceptability testing. JMIR Res Protoc.

[94] Holtz B, Murray KM, Hershey DD, Dunneback JK, Cotten SR, Holmstrom AJ, Vyas A, Kaiser MK, Wood MA (2017). Developing a patient-centered mHealth app: a tool for adolescents with type 1 diabetes and their parents. JMIR Mhealth Uhealth.

[95] Fishbein JN, Nisotel LE, MacDonald JJ, Amoyal Pensak N, Jacobs JM, Flanagan C, Jethwani K, Greer JA (2017). Mobile application to promote adherence to oral chemotherapy and symptom management: a protocol for design and development. JMIR Res Protoc.

[96] Gabrielli S, Dianti M, Maimone R, Betta M, Filippi L, Ghezzi M, Forti S (2017). Design of a mobile app for nutrition education (TreC-LifeStyle) and formative evaluation with families of overweight children. JMIR Mhealth Uhealth.

[97] Jibb L, Cafazzo JA, Nathan PC, Seto E, Stevens BJ, Nguyen C, Stinson JN (2017). Development of a mHealth real-time pain self-management app for adolescents with cancer: an iterative usability testing study [Formula: see text]. J Pediatr Oncol Nurs.

[98] Stephan LS, Dytz AE, Guimaraes RB, Ley AG, Mathias RG, Assis MV, Leiria TLL (2017). Processes and Recommendations for Creating mHealth Apps for Low-Income Populations. JMIR Mhealth Uhealth.

[99] Chen J, Lieffers J, Bauman A, Hanning R, Allman-Farinelli M (2017). Designing health apps to support dietetic professional practice and their patients: qualitative results from an international survey. JMIR Mhealth Uhealth.

[100] Mishori R, Anastario M, Naimer K, Varanasi S, Ferdowsian H, Abel D, Chugh K (2017). mJustice: preliminary development of a mobile app for medical-forensic documentation of sexual violence in low-resource environments and conflict zones. Glob Health Sci Pract.

[101] Docking R, Lane Matthew, Schofield Pat A (2018). Usability Testing of the iPhone App to Improve Pain Assessment for Older Adults with Cognitive Impairment (Prehospital Setting): A Qualitative Study. Pain Med.

[102] Tay I, Garland S, Gorelik A, Wark JD (2017). Development and testing of a mobile phone app for self-monitoring of calcium intake in young women. JMIR Mhealth Uhealth.

[103] López D, Torres M, Vélez J, Grullon J, Negrón E, Pérez CM, Palacios C (2017). Development and evaluation of a nutritional smartphone application for making smart and healthy choices in grocery shopping. Healthc Inform Res.

[104] Armin J, Johnson T, Hingle M, Giacobbi P, Gordon JS (2017). Development of a multi-behavioral mHealth app for women smokers. J Health Commun.

[105] Zhu J, Ebert L, Xue Z, Shen Q, Chan SW (2017). Development of a mobile application of Breast Cancer e-Support program for women with breast cancer undergoing chemotherapy. Technol Health Care.

[106] Cerrada CJ, Dzubur E, Blackman KC, Mays V, Shoptaw S, Huh J (2017). Development of a Just-in-Time adaptive intervention for smoking cessation among Korean American emerging adults. Int J Behav Med.

[107] Rickard N, Arjmand HA, Bakker D, Seabrook E (2016). Development of a mobile phone app to support self-monitoring of emotional well-being: a mental health digital innovation. JMIR Ment Health.

[108] Gordon JS, Armin JS, Cunningham JK, Muramoto ML, Christiansen SM, Jacobs TA (2017). Lessons learned in the development and evaluation of RxCoach™, an mHealth app to increase tobacco cessation medication adherence. Patient Educ Couns.

[109] Chen S, Gong E, Kazi DS, Gates AB, Karaye KM, Girerd N, Bai R, AlHabib KF, Li C, Sun K, Hong L, Fu H, Peng W, Liu X, Chen L, Schwalm J, Yan LL (2016). Development of a mobile phone-based intervention to improve adherence to secondary prevention of coronary heart disease in China. J Med Eng Technol.

[110] Buman MP, Epstein DR, Gutierrez M, Herb C, Hollingshead K, Huberty JL, Hekler EB, Vega-López S, Ohri-Vachaspati P, Hekler AC, Baldwin CM (2016). BeWell24: development and process evaluation of a smartphone “app” to improve sleep, sedentary, and active behaviors in US Veterans with increased metabolic risk. Transl Behav Med.

[111] Mummah S, King AC, Gardner CD, Sutton S (2016). Iterative development of Vegethon: a theory-based mobile app intervention to increase vegetable consumption. Int J Behav Nutr Phys Act.

[112] Yardley L, Morrison L, Bradbury K, Muller I (2015). The person-based approach to intervention development: application to digital health-related behavior change interventions. J Med Internet Res.

[113] Van Lippevelde W, Vangeel J, De Cock N, Lachat C, Goossens L, Beullens K, Vervoort L, Braet C, Maes L, Eggermont S, Deforche B, Van Camp J (2016). Using a gamified monitoring app to change adolescents' snack intake: the development of the REWARD app and evaluation design. BMC Public Health.

[114] White B, Martin A, White JA, Burns SK, Maycock BR, Giglia RC, Scott JA (2016). Theory-based design and development of a socially connected, gamified mobile app for men about breastfeeding (Milk Man). JMIR Mhealth Uhealth.

[115] Vorrink S, Kort HS, Troosters T, Lammers JW (2016). A mobile phone app to stimulate daily physical activity in patients with chronic obstructive pulmonary disease: development, feasibility, and pilot studies. JMIR Mhealth Uhealth.

[116] Panatto D, Domnich A, Gasparini R, Bonanni P, Icardi G, Amicizia D, Arata L, Bragazzi NL, Signori A, Landa P, Bechini A, Boccalini S (2016). Development and preliminary data on the use of a mobile app specifically designed to increase community awareness of invasive pneumococcal disease and its prevention. Hum Vaccin Immunother.

[117] Athilingam P, Osorio R, Kaplan H, Oliver D, O'neachtain T, Rogal P (2016). Embedding patient education in mobile platform for patients with heart failure: theory-based development and beta testing. Comput Inform Nurs.

[118] Mann D, Riddell L, Lim K, Byrne LK, Nowson C, Rigo M, Szymlek-Gay EA, Booth AO (2015). Mobile phone app aimed at improving iron intake and bioavailability in premenopausal women: a qualitative evaluation. JMIR Mhealth Uhealth.

[119] Jaensson M, Dahlberg K, Eriksson M, Grönlund Å, Nilsson U (2015). The development of the recovery assessments by phone points (RAPP): a mobile phone app for postoperative recovery monitoring and assessment. JMIR Mhealth Uhealth.

[120] Boyd A, Moores K, Shah V, Sadhu E, Shroff A, Groo V, Dickens C, Field J, Baumann M, Welland B, Gutowski G, Flores JD, Zhao Z, Bahroos N, Hynes DM, Wilkie DJ (2015). My Interventional Drug-Eluting Stent Educational App (MyIDEA): patient-centered design methodology. JMIR Mhealth Uhealth.

[121] Juarascio A, Goldstein SP, Manasse SM, Forman EM, Butryn ML (2015). Perceptions of the feasibility and acceptability of a smartphone application for the treatment of binge eating disorders: Qualitative feedback from a user population and clinicians. Int J Med Inform.

[122] Knight-Agarwal C, Davis DL, Williams L, Davey R, Cox R, Clarke A (2015). Development and pilot testing of the Eating4two mobile phone app to monitor gestational weight gain. JMIR Mhealth Uhealth.

[123] Lim J, Cloete G, Dunsmuir DT, Payne BA, Scheffer C, von Dadelszen P, Dumont GA, Ansermino JM (2015). Usability and feasibility of PIERS on the move: an mHealth app for pre-eclampsia triage. JMIR Mhealth Uhealth.

[124] Revenäs Å, Opava CH, Martin C, Demmelmaier I, Keller C, Åsenlöf P (2015). Development of a web-based and mobile app to support physical activity in individuals with rheumatoid arthritis: results from the second step of a co-design process. JMIR Res Protoc.

[125] Hilliard M, Hahn A, Ridge AK, Eakin MN, Riekert KA (2014). User preferences and design recommendations for an mHealth app to promote cystic fibrosis self-management. JMIR Mhealth Uhealth.

[126] Mirkovic J, Kaufman DR, Ruland CM (2014). Supporting cancer patients in illness management: usability evaluation of a mobile app. JMIR Mhealth Uhealth.

[127] O'Malley G, Dowdall G, Burls A, Perry IJ, Curran N (2014). Exploring the usability of a mobile app for adolescent obesity management. JMIR Mhealth Uhealth.

[128] Hearn L, Miller M, Lester L (2014). Reaching perinatal women online: the Healthy You, Healthy Baby website and app. J Obes.

[129] de la Vega R, Roset R, Castarlenas E, Sánchez-Rodríguez E, Solé E, Miró J (2014). Development and testing of painometer: a smartphone app to assess pain intensity. J Pain.

[130] van Velsen Lex, Beaujean DJ, Wentzel J, Van Steenbergen JE, van Gemert-Pijnen JE (2015). Developing requirements for a mobile app to support citizens in dealing with ticks and tick bites via end-user profiling. Health Informatics J.

[131] van der Weegen S, Verwey R, Spreeuwenberg M, Tange H, van der Weijden T, de Witte L (2013). The development of a mobile monitoring and feedback tool to stimulate physical activity of people with a chronic disease in primary care: a user-centered design. JMIR Mhealth Uhealth.

[132] Sockolow P, Schug S, Zhu J, Smith TJ, Senathirajah Y, Bloom S (2017). At-risk adolescents as experts in a new requirements elicitation procedure for the development of a smart phone psychoeducational trauma-informed care application. Inform Health Soc Care.

[133] DeLaughter K, Sadasivam RS, Kamberi A, English TM, Seward GL, Chan SW, Volkman JE, Amante DJ, Houston TK (2016). Crave-Out: a distraction/motivation mobile game to assist in smoking cessation. JMIR Serious Games.

[134] Alnasser A, Sathiaseelan A, Al-Khalifa A, Marais D (2016). Development of 'Twazon': an Arabic app for weight loss. JMIR Res Protoc.

[135] LeGrand S, Muessig KE, McNulty T, Soni K, Knudtson K, Lemann A, Nwoko N, Hightow-Weidman LB (2016). Epic Allies: development of a gaming app to improve antiretroviral therapy adherence among young HIV-positive men who have sex with men. JMIR Serious Games.

[136] Heffernan K, Chang S, Maclean ST, Callegari ET, Garland SM, Reavley NJ, Varigos GA, Wark JD (2016). Guidelines and recommendations for developing interactive eHealth apps for complex messaging in health promotion. JMIR Mhealth Uhealth.

[137] Albornoz MA, Márquez S, Rubin L, Luna D (2017). Design of a mobile application for transfusion medicine. Stud Health Technol Inform.

[138] Martinez B, Hall-Clifford R, Coyote E, Stroux L, Valderrama CE, Aaron C, Francis A, Hendren C, Rohloff P, Clifford GD (2017). Agile development of a smartphone app for perinatal monitoring in a resource-constrained setting. J Health Inform Dev Ctries.

[139] Ehrler F, Lovis C, Blondon K (2016). Addressing the complexity of mobile app design in hospital setting with a tailored software development life cycle model. Stud Health Technol Inform.

[140] Crosby L, Ware RE, Goldstein A, Walton A, Joffe NE, Vogel C, Britto MT (2017). Development and evaluation of iManage: a self-management app co-designed by adolescents with sickle cell disease. Pediatr Blood Cancer.

[141] Schnall R, Rojas M, Bakken S, Brown W, Carballo-Dieguez A, Carry M, Gelaude D, Mosley JP, Travers J (2016). A user-centered model for designing consumer mobile health (mHealth) applications (apps). J Biomed Inform.

[142] Wilhide Iii CC, Peeples MM, Anthony Kouyaté RC (2016). Evidence-based mHealth chronic disease mobile app intervention design: development of a framework. JMIR Res Protoc.

[143] Goyal S, Morita P, Lewis GF, Yu C, Seto E, Cafazzo JA (2016). The systematic design of a behavioural mobile health application for the self-management of type 2 diabetes. Can J Diabetes.

[144] Curtis K, Lahiri S, Brown KE (2015). Targeting parents for childhood weight management: development of a theory-driven and user-centered healthy eating app. JMIR Mhealth Uhealth.

[145] Mummah S, Robinson TN, King AC, Gardner CD, Sutton S (2016). IDEAS (Integrate, Design, Assess, and Share): a framework and toolkit of strategies for the development of more effective digital interventions to change health behavior. J Med Internet Res.

[146] Whittaker R, Merry S, Dorey E, Maddison R (2012). A development and evaluation process for mHealth interventions: examples from New Zealand. J Health Commun.

[147] Van Velsen L, Wentzel J, Van Gemert-Pijnen JE (2013). Designing eHealth that matters via a multidisciplinary requirements development approach. JMIR Res Protoc.

[148] Hevner AR (2007). Scand J Inform Syst.

[149] Graham ID, Logan J, Harrison MB, Straus SE, Tetroe J, Caswell W, Robinson N (2006). Lost in knowledge translation: time for a map?. J Contin Educ Health Prof.

[150] Collins LM, Baker TB, Mermelstein RJ, Piper ME, Jorenby DE, Smith SS, Christiansen BA, Schlam TR, Cook JW, Fiore MC (2011). The multiphase optimization strategy for engineering effective tobacco use interventions. Ann Behav Med.

[151] Michie S, van Stralen MM, West R (2011). The behaviour change wheel: a new method for characterising and designing behaviour change interventions. Implement Sci.

[152] Glanz K, Rimer BK, Viswanath K (2008). Health Behavior and Health Education: Theory, Research, and Practice.

[153] Raiff B, Fortugno N, Scherlis D, Rapoza D (2018). A mobile game to support smoking cessation: prototype assessment. JMIR Serious Games.

[154] Bauckhage C, Drachen A, Sifa R (2015). Clustering Game Behavior Data. IEEE Trans Comput Intell AI Games.

